# Music Proficiency and Quantification of Absolute Pitch: A Large-Scale Study among Brazilian Musicians

**DOI:** 10.3389/fnins.2016.00447

**Published:** 2016-10-13

**Authors:** Raphael B. C. Leite, Sergio A. Mota-Rolim, Claudio M. T. Queiroz

**Affiliations:** Brain Institute, Federal University of Rio Grande do NorteNatal, Brazil

**Keywords:** pitch perception, absolute pitch, music proficiency, pitch-naming test, pitch class, perfect pitch, pitch identification

## Abstract

Absolute pitch (AP) is the ability to identify and name the pitch of a sound without external reference. Often, accuracy and speed at naming isolated musical pitches are correlated with demographic, biological, and acoustical parameters to gain insight into the genesis and evolution of this ability in specific cohorts. However, the majority of those studies were conducted in North America, Europe, or Asia. To fill this gap, here we investigated the pitch-naming performance in a large population of Brazilian conservatory musicians (*N* = 200). As previously shown, we found that the population performance was rather a continuum than an “*all-or-none*” ability. By comparing the observed distribution of correct responses to a theoretical binomial distribution, we estimated the prevalence of AP as being 18% amongst regular music students. High accuracy thresholds (e.g., 85% of correct responses) yielded a prevalence of 4%, suggesting that AP might have been underestimated in previous reports. Irrespective of the threshold used, AP prevalence was higher in musicians who started their musical practice and formal musical education early in life. Finally, we compared the performance of those music students (average proficiency group) with another group of students selected to take part in the conservatory orchestra (high proficiency group, *N* = 30). Interestingly, the prevalence of AP was higher in the latter in comparison to the former group. In addition, even when the response was incorrect, the mean absolute deviation from the correct response was smaller in the high proficiency group compared to the average proficiency group (Glass's Δ: 0.5). Taken together, our results show that the prevalence of AP in Brazilian students is similar to other non-tonal language populations, although this measure is highly dependent on the scoring threshold used. Despite corroborating that early involvement with musical practice and formal education can foster AP ability, the present data suggest that music proficiency may also play an important role in AP expression.

## Introduction

Evolution of auditory and vocal systems has reached high levels of complexity in humans, allowing the emergence of both speech and music (Patel, 2003). In this respect, one important feature of sound is pitch, which grants prosody to spoken speech and melody to music. Although, pitch perception is ubiquitous in the animal kingdom (Weisman et al., 2004), some individuals are able to identify and name the pitch of a particular sound without any external reference, a supposedly rare ability known as absolute pitch (AP) or perfect pitch (Bachem, 1937; Takeuchi and Hulse, 1993; Levitin and Rogers, 2005; Deutsch, 2013).

The prevalence of AP is highly heterogeneous and culture-specific, as it relies on how signifiers (pitch label) and signified (periodicity of sound waves) relates to each other (Nattiez, 1990). Considering Western populations in Europe and North America, the prevalence of AP has been estimated between 0.01% (Bachem, 1955) and 0.07% (Profita and Bidder, 1988). However, the methodology of these early studies is hard to replicate and include biased samples and subjective measures. It is now acknowledged that the prevalence of AP can be as high as 75% among specific cohorts, as in music schools and conservatories (Baharloo et al., 1998; Gregersen, 1999; Deutsch et al., 2006, 2009; Miyazaki et al., 2012; see also Sergeant and Vraka, 2014).

It has been proposed that contrasting AP prevalence among different populations could be explained by the influence of genes (Baharloo et al., 1998; Gregersen, 1999; Theusch and Gitschier, 2011) and/or environmental constrains (Baharloo et al., 1998; Miyazaki et al., 2012; Wilson et al., 2012; Deutsch, 2013). In this respect, different hypotheses were proposed to explain AP development and expression (for a review, see Takeuchi and Hulse, 1993; Deutsch, 2013). The first hypothesis suggests a correlation between genes and the cognitive ability to listen to pitches in absolute terms, initially put forward by pedigree analysis that found a higher prevalence of AP among first-degree relatives (Baharloo et al., 1998; Profita and Bidder, 1988), siblings (Baharloo et al., 2000), and twins (Gregersen, 1998). The higher prevalence among Asian in comparison to Caucasian subjects was also considered as an indirect evidence for the genetic predisposition for AP (Gregersen et al., 2001), although cultural factors cannot be ruled out (see the second hypothesis below). Despite these evidences, sophisticated whole-genome linkage analyzes failed to reveal specific AP-related genes, which prompts the conclusion that AP may be genetically heterogeneous (Theusch et al., 2009). Therefore, pinpointing specific genes related to the predisposition to AP is now seen with skepticism, and alternative hypotheses need to be considered.

A second hypothesis proposes that full acquisition of AP ability can only take place when subjects are taught to relate pitch and musical labels within the “sensitive period” of development (Deutsch, 2013). The *early learning hypothesis* proposes that internalization of sound and its association with a specific label relies on the increased plasticity governing the formation of neural assemblies early in life (Deutsch, 2013). In fact, AP prevalence is higher among musicians who started their formal musical education before 6 years old (Baharloo et al., 1998; Deutsch et al., 2006; Miyazaki et al., 2012). Nonetheless, some studies have reported improved performance after extensive training in both children (Crozier, 1997; Russo et al., 2003; Miyazaki and Ogawa, 2006) and adult populations (Vianello and Evans, 1968; Brady, 1970; Heller and Auerbach, 1972; Van Hedger et al., 2015).

Finally, the third hypothesis considers the musical training regime and/or environment. For example, it has been shown that the prevalence of AP can be higher among musicians who speak tonal languages in comparison to non-tonal language speakers (Deutsch et al., 2006, 2009, 2013). In this case, the cognitive demand to extract meaning from similar sounds with varied intonations would extrapolate to other tasks, and therefore, contribute to pitch recognition and naming. However, early music education policies in Asian countries may also contribute to the differences in the observed prevalence (Miyazaki and Ogawa, 2006; Miyazaki et al., 2012), as music education methods (i.e., “fixed-do” vs. “moveable-do”) and the type of instrument used in learning classes (Miyazaki et al., 2012; Deutsch et al., 2013). In this respect, at least to our knowledge, few studies have attempted to investigate the influence of music proficiency in the expression of AP, which may have been overlooked in previous reports.

Music is one of the most important aspects of Brazilian identity. Some rhythmic and harmonic styles of the Brazilian music, like choro, samba, and bossa-nova, are well-known for its originality and diversity (McGowan and Pessanha, 1998). Interestingly, most Brazilian musicians start their music education informally (Feichas, 2010) and governmental support is scarce. Together, those conditions restrict the access to formal educational institutions to the most “gifted” musicians. In the present study, we used a pitch-naming task (Deutsch et al., 2006) in a large population of conservatory students to determine the prevalence of AP in a cohort of Brazilian musicians. Early studies using self-reported questionnaires observed an AP prevalence of 5% among Brazilian music students (Germano et al., 2011, 2013). However, these results could be biased since musicians may covet this ability, and to overcome such limitation, psychophysical tests are mandatory (Bermudez and Zatorre, 2009). The methodologies of psychophysical tests often yield varied degrees of performance at the population level raising another relevant question: is there a clear threshold to separate AP possessors from non-AP possessors? Curiously, this threshold is set arbitrarily in most studies. Here, we try to overcome the threshold problem by statistically comparing individual performance in a pitch-naming test with a theoretical binomial distribution. This approach allowed the identification of AP possessors whose performance surpassed chance. For comparison purposes, we also included a sub maximal threshold used in previous reports (Deutsch et al., 2006, 2009, 2013). Finally, we analyze and present our results in light of aforementioned hypothesis—characterizing our sample according to (1) the age of onset of musical training and formal musical education, (2) musical instrument, and (3) musical proficiency. This third factor was evaluated by comparing the prevalence of AP among regular music students (average proficiency group) and those selected to take part in the conservatory orchestra (high proficiency group). To our knowledge, this is the first empirical study aimed to determine the prevalence and characteristics of AP in Latin America.

## Materials and methods

### Participants

Students from the Music School of the Federal University of Rio Grande do Norte were approached in the beginning of the academic year (February and March 2016) and were invited to take part in an experiment related to music perception. Thirteen out of 41 courses allowed the application of the test. A total of 200 regular students (55 female, 24 ± 7 years-old; 145 male, 25 ± 9 years-old) satisfied the inclusion criteria (reported no auditory or neurological disorders) and fully completed all stages of the test. Participants did not receive any financial support (as dictated by Brazilian regulations) and provided written informed consent before the experiments were realized, in compliance with international standards. All protocols and procedures were previously approved by the Ethics Committee of the Federal University of Rio Grande do Norte (CEP-UFRN #31273114.5.0000.5537).

To further investigate the role of musical proficiency in the prevalence of AP, a second group of high proficiency music students were included in the test (*N* = 30; 4 female, 21 ± 4 years-old; 26 male, 24 ± 5 years-old). This group represents especially talented students who have succeeded at a high-level orchestral audition, in which the musicians perform musical pieces belonging to the orchestral repertoire. The audition is judged by teachers from the conservatory, as well as by an expert in the musician's instrument. Besides the social visibility within the conservatory and the positive impact in their careers, the selected students are entitled to receive scholarships. For these reasons, the competition to fulfill these positions is high and only high proficiency musicians are selected.

### Questionnaire

Before performing the pitch-naming task, participants were asked to complete a questionnaire informing their age, history of musical education (start and duration of both informal and formal musical training), training habits (schedule and estimated amount of time practicing), and instruments played. These questions aimed to investigate the contribution of musical education during sensitive periods.

### Stimuli

The set of stimuli consisted of 36 piano tones (1 s duration), ranging from C3 (131 Hz) to B5 (988 Hz), synthetically generated (GarageBand, Apple Inc.). The harmonic sequence consisted of the fundamental frequency and its harmonics conferring a musical piano timbre to the synthetic sound. The stimuli were presented through two loudspeakers (~70 dB) with the assistance of a personal computer running SuperLab 4.5 software (Cedrus Corporation, Inc.).

### Pitch identification test

The pitch-naming task used was modified from a previous report (Deutsch et al., 2006). A set of 36 stimuli was presented randomly and repeated once. Each set consisted of three blocks of 12 stimuli, with an interval of 30 s between blocks to minimize fatigue. Before starting the test, we presented a training block with four trials. Participants were asked to write down the name of each note as fast as possible. The interval between stimuli was set to 4.25 s. To avoid the use of strategies based on relative pitch judgments, the tonal distance between each tone sequentially presented was higher than one octave, and never had one semitone interval (not considering the octave). No octave information was requested and no feedback was given, neither during the training, nor during the test blocks. The tests were conducted in the classrooms and in the conservatory theater for the regular and orchestra groups, respectively.

### Data analysis and statistics

The percentage of correct responses was calculated by scoring the exact identification of pitch. Thus, no semitone errors were allowed as even small deviations in pitch identification violate the general meaning of AP. Besides the criterion of pitch distance, AP prevalence also depends on the threshold used to infer the percentage of AP possessors. We formed two different criteria for AP, due to the lack of consensus in the literature (Gregersen, 1999; Athos et al., 2007; Miyazaki et al., 2012; Deutsch et al., 2013). The first one, defined as *conservative* AP (cAP), included subjects scoring at least 85% of correct answers. This threshold was based on previous publications (see Deutsch et al., 2009, 2006) and it was selected to allow comparisons between the present work and similar previous large-scale studies. The second threshold considers the identification of AP possessors in the population from a statistical point of view (Sergeant and Vraka, 2014). Thus, the chance expectation can be determined by the binomial distribution, which calculates the likelihood *P*_*k*_ of observing *k* correct choices in *n* number of trials, given the probability *p* in each trial, according to the equation:

$$\begin{array}{l} P_{k}=\left(\frac{n!}{k!\left(n-k\right)!}\right)p^{k}\;{\left(1-p\right)}^{n-k} \end{array}$$

Considering 72 trials and the probability of correct response in each trial to be 1:12, the mean probability of correct responses was calculated as 6 correct responses (i.e., 8.3%). We used the Clopper–Pearson method (Clopper and Pearson, 1934; *binofit* function in Matlab) with 99% confidence interval to determine the threshold of significance to regard the performance above chance. Here, the first integer value outside this confidence interval was 15 (i.e., 20%), and this value was considered the threshold for our *inclusive* AP (iAP) approach. Therefore, participants with 15 or more correct responses, but less than 61 (i.e., 85%) were considered as presenting some level of absolute pitch ability, and treated separately from the cAP group.

Mean absolute deviation (MAD), with and without correct trials, was computed and correlated with the performance (correct responses yields absolute deviation = 0, while one semitone mistake yields absolute deviation = 1). Correlation between the first and second block was calculated to control for changes in performance during the test (Bermudez and Zatorre, 2009). Correct responses and MAD were used to compare the effects of pitch class and pitch label. Differences in frequency distributions of categorical variables were compared using Pearson's chi-squared test. We used one-way analysis of variance (ANOVA), followed by multiple comparison *post-hoc* Bonferroni test, to compare independent variables (age, amount of daily hours practicing with musical instruments in the last year, mean absolute deviation, and performance). Welch's *F*-test was used in case the homogeneity of variance assumption was violated. In some comparisons, effect size was calculated using Glass's Δ method. Data are presented as mean ± standard deviation, and a *p*-value smaller than 0.05 was considered of statistical significance in all comparisons made.

## Results

### Prevalence of AP ability

**Figure 1:**
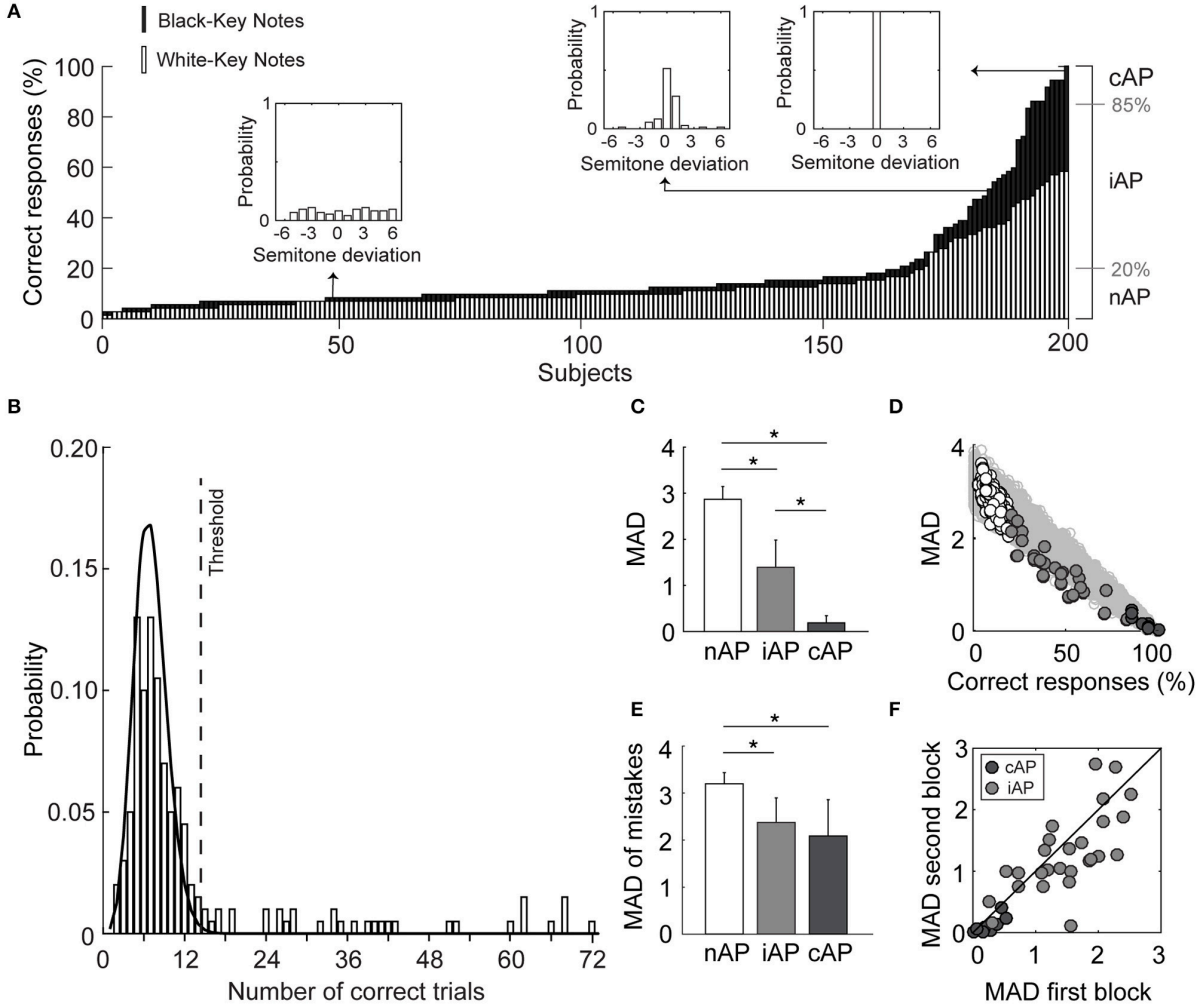
**Quantification of Absolute Pitch (AP) demonstrates that population performance is a continuum rather than an “all-or-none” ability. (A)** Ranked performance (each bar represents one subject) shows that subjects with low performance identify white-key notes with more accuracy than black-key notes. Right of the graph shows the representation of the conservative (cAP) and inclusive (iAP) thresholds used for separating populations. Insets represent the histogram of the distances from correct responses to the presented stimuli. **(B)** The chance of correctly naming the pitch was calculated using a binomial distribution (solid line). The histogram represents the observed performance distribution. The dashed line represents the 99% confidence interval for chance-level scores (35 subjects, 18%, were above threshold). **(C)** Mean absolute deviation (MAD, in semitones) for each of the three groups. **(D)** Scatter plot showing the correlation between percentage of correct responses and mean absolute deviation. Note that the observed data do not fit in shaded area (simulated data), mainly for the iAP group. **(E)** Mean absolute deviation of incorrect responses. Note that iAP group responded closer to correct pitch than the nAP group. **(F)** Consistent responses of iAP and cAP were verified by correlating the mean absolute deviation between the first and the second blocks of 36 trials. ^*^p < 0.05, *t*-test.

The pitch-naming test revealed that performance varied significantly in our cohort of music students (Figure 1). It also reiterated that the population performance is a continuum rather than a discrete bimodal ability (Figure 1A). This poses the challenging decision in determining the threshold to classify a participant as AP possessor, which in turn, influences the prevalence of the ability. Using the threshold of 85% of exactly correct responses (Deutsch et al., 2006), we observed a prevalence of 4% (cAP group). The inclusive criteria (iAP), based on a binomial distribution, instead yielded a prevalence of 14% (Figure 1B). Together, cAP and iAP groups made 18% of our cohort (35 out of 200 subjects) while the others (non-AP; nAP) performed similarly to chance. The prevalence of AP using the self-reported questionnaire returned similar values (3.5%) to the cAP group, but surprisingly the participants who reported having the ability were not the same of those who surpassed the threshold (conservative and inclusive) on the pitch identification test (Table 1). The false-positive and false-negative rates were 2.5% (4/162) and 90% (29/32), respectively, when considering the self-reported questionnaire as the predictive condition. It is important to note that most of the false-negative subjects belong to the iAP group (24/29). No significant differences were observed in the prevalence of AP among male and female participants (Male: 19% and Female: 15%; *p* > 0.05, chi-square test), age at the time of the test [in years-old; nAP: 26 ± 9, iAP: 22 ± 6 and cAP: 24 ± 7; *F*_(2, 197)_ = 2.79, *p* > 0.05, one-way ANOVA], and the amount of daily hours practicing with musical instruments in the last year [in hours; nAP: 2.5 ± 1.7, iAP: 2.9 ± 1.5, and cAP: 2.8 ± 2.3; *F*_(2, 197)_ = 0.64, *p* > 0.05, one-way ANOVA].

**Table 1:** **Contingency table of self-reported AP ability per group of psychophysically identified AP possessors or non-AP possessors**.

**Psychophysical test**	**Self-reported AP**			
	**Yes**	**No**	**NR^♢^**	**Total**
Yes^§^	3	29	3	35
No	4	158	3	165
Total	7	187	6	200

♢ NR, Non Respondent;

§ *Yes, iAP + cAP*.

To determine whether the iAP group constituted a different population from the nAP group, we compared the mean absolute deviation between groups. The smallest and highest mean absolute deviation values were observed for the cAP and nAP groups, respectively, while iAP group showed intermediate values [*F*_(2, 18.53)_ = 1047.30, *p* < 0.001; Welch's *F*-test; Glass's estimator of effect size between cAP and iAP = 2.03, and between iAP and nAP = 5.28; Figure 1C]. However, this was not surprising since mean absolute deviation and percentage of correct responses—the variable that we used to classify the groups—are strongly correlated (*R*^2^ = 0.90, *p* < 0.001; Figure 1D). To better understand the distribution of the mean absolute deviation in our cohort, we simulated the values of the mean absolute deviations (using uniform distributions) for each possible performance value (200 iterations per score). The gray-shaded area in Figure 1D shows the simulated data and how our sample downwardly dispersed from it. This observation suggests that as the performance increases, the pitch distance between the response and stimulus decays faster. To test whether the distances of responses to the stimuli were indeed smaller in the iAP group in comparison to the nAP group, we recalculated the mean absolute deviations excluding the correct responses (i.e., absolute deviation = 0; Figure 1E). This approach allowed the demonstration that iAP group indeed differs from nAP group, once the former guessed more closely the stimulus pitch even when making wrong choices [*F*_(2, 13.24)_ = 37.58; *p* < 0.05; Welch's *F*-test; Glass's estimator of effect size between cAP and iAP = 0.55, and between iAP and nAP = 3.46]. Finally, we tested the consistency of the responses for the AP groups by correlating the mean absolute deviations in the first and in the second blocks of stimuli (Figure 1F). We observed a positive correlation between the blocks (*R*^2^ = 0.69, *p* < 0.001; Pearson test), although most of the subjects (mainly from the cAP group) improved their performance during the test, as revealed by an asymmetrical distribution of points above and below the diagonal line (which represents the equal performance between blocks; Figure 1F).

### Contribution of pitch class to the performance

**Figure 2:**
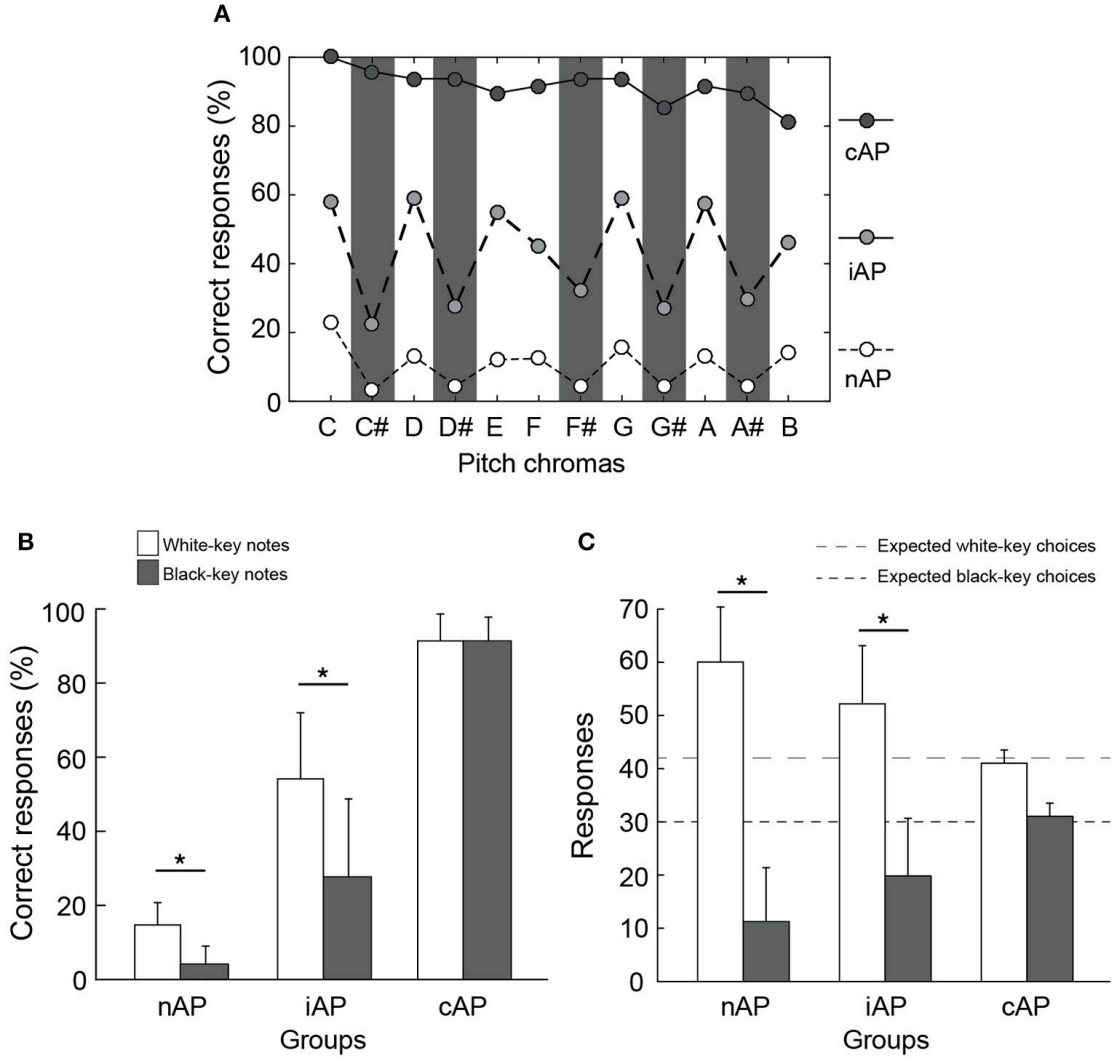
**Participants of nAP and iAP groups identified white-key notes better than black-key notes. (A)** Performance of each pitch chroma for each group of participants. The performances for black-key notes (sharp/flat) are represented in a gray background. **(B)** Averaged correct responses according to pitch class (i.e., white-key and black-key notes). In the nAP and iAP groups, the performance for white-key notes was higher than for black-key notes ^*^p < 0.001, paired-*t*-test. **(C)** Averaged white-key and black-key notes responses, irrespective of whether they were correct or incorrect. Dashed lines represent the expected white-key and black-key notes responses according to the set stimuli. ^*^p < 0.001, chi-square test.

Performance for natural (a.k.a., white-key) and accidental (a.k.a., black-key) notes varied in our population, mainly in the iAP group (Figure 2A). Interestingly, nAP group, whose performance was below chance, clearly fetched more white-key notes than black-key notes [Figure 2B; *t*_(164)_ = 16.45, *p* < 0.001, paired *t*-test; Figure 2C; chi-square for observed vs. expected responses: $\chi_{\left(1,\;11752\right)}^{2}$ = 3216, *p* < 0.001]. Similar unbalanced performance for white-key notes was also observed in iAP group [*t*_(26)_ = 7.30, *p* < 0.001, paired *t*-test; Figure 2C; chi-square for observed vs. expected responses: $\chi_{\left(1,\;1942\right)}^{2}$ = 160, *p* < 0.001]. However, no difference between white-key and black-key notes was observed in the cAP group.

### Onset of musical training and formal musical education

**Figure 3:**
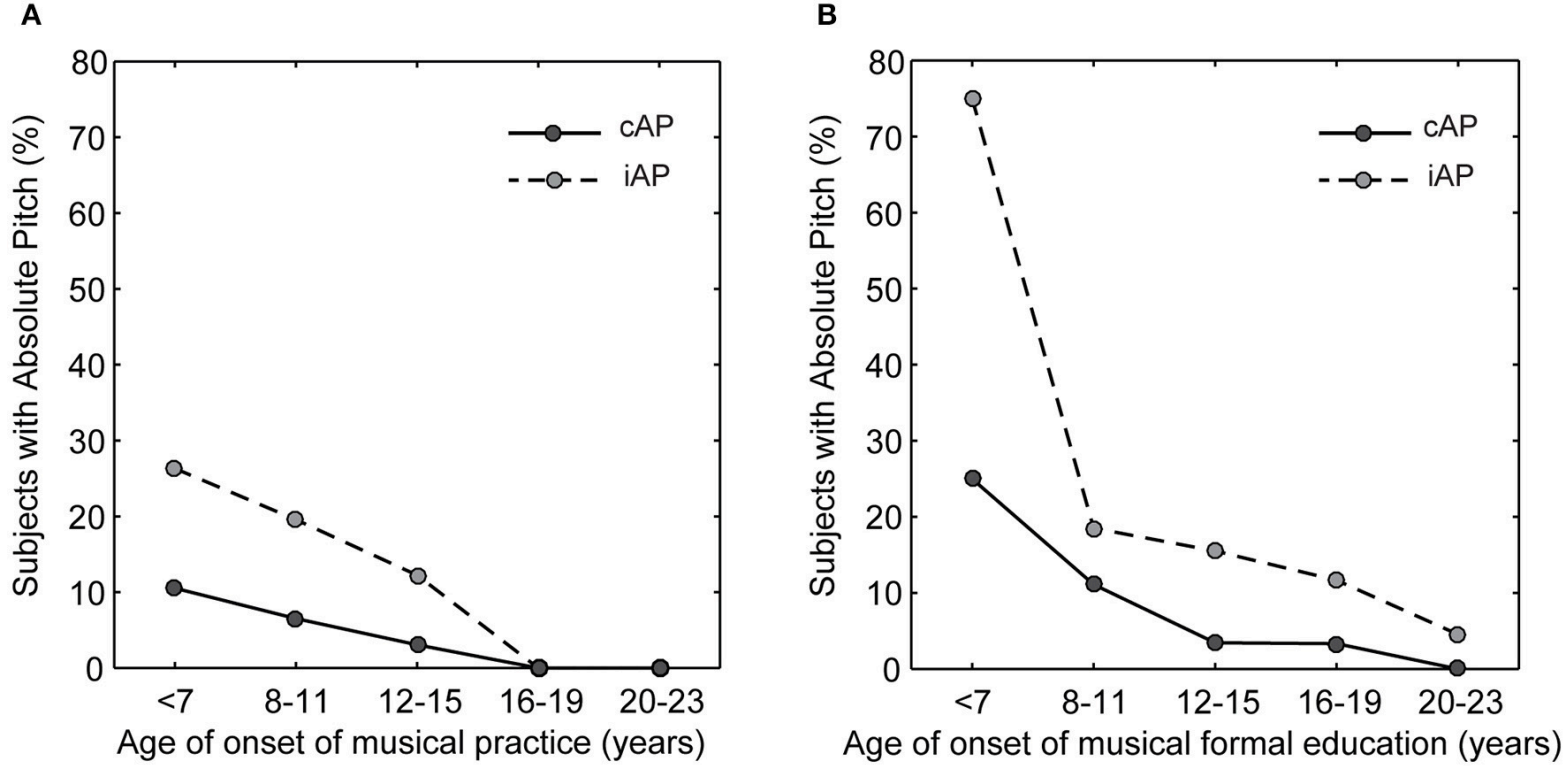
**Absolute pitch has a higher prevalence in musicians who started musical training early in life**. Percentages of AP possessors according to the **(A)** age of onset (in years) of musical practice, and **(B)** age of onset (in years) of formal music education. Solid lines represent subjects of the cAP group, and dashed lines represent subjects of the iAP group.

To investigate the relationship between AP and the early onset of musical training, as previously reported in the literature, we divided participants in bins of age (years-old) of onset in both informal (<7: *N* = 19, 8–11: *N* = 46, 12–15: *N* = 98, 16–19: *N* = 28, 20–23: *N* = 4) and formal (<7: *N* = 4, 8–11: *N* = 27, 12–15: *N* = 58, 16–19: *N* = 60, 20–23: *N* = 22) musical practice. We observed a higher prevalence of AP in musicians who started their musical practice early in life (Figure 3). This was true when considering both the onset of musical practice (Figure 3A) and formal musical education (Figure 3B). While the latter probably involves classes of musical theory and some sort of organized methodology (not investigated here), the former deals with informal and probably exclusively “playing by ear” approach of learning pitch.

### Instrument class

**Figure 4:**
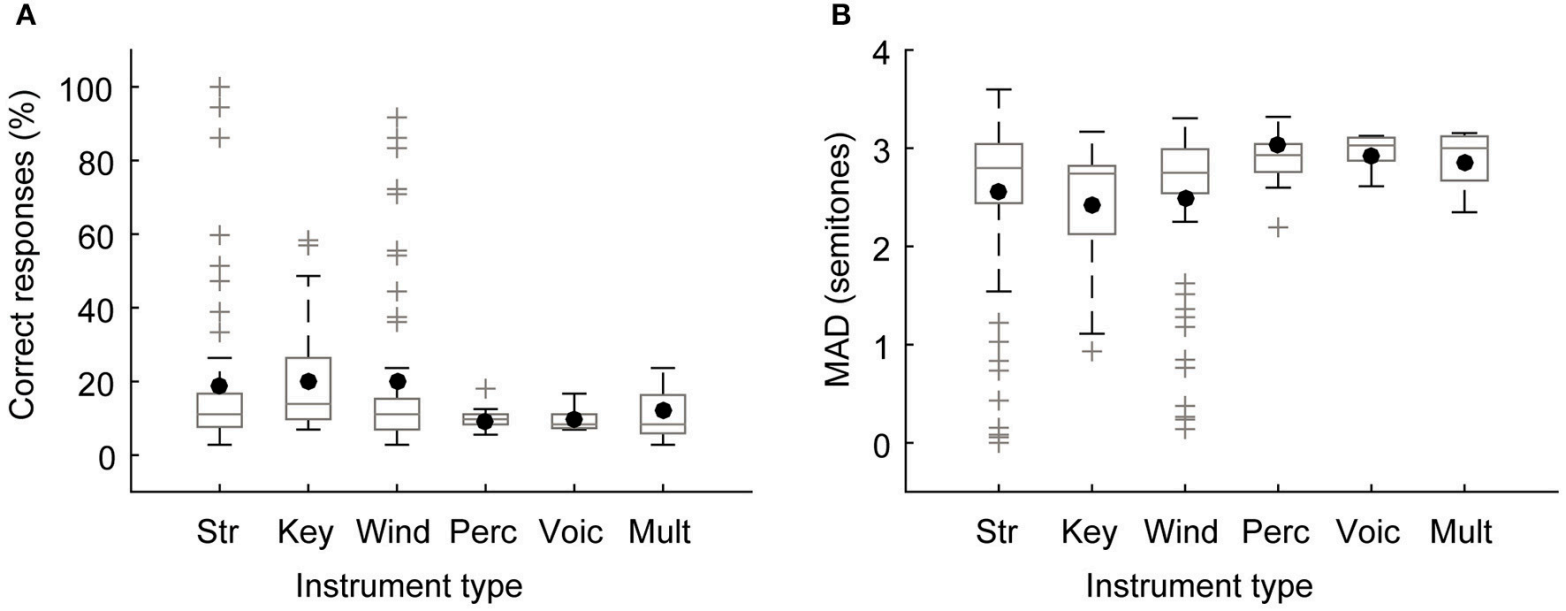
**Pitch identification performance does not depend on the instrument played by the subjects. (A)** Performance and **(B)** mean absolute deviation. Box plots of the distribution scores represent the median (horizontal line), the interquartile range (box width), and the outliers (gray crosses). The black dots represent the mean. Str, strings; Key, keyboards; Wind, winds; Perc, percussions; Voic, voice, and Mult, multi-instruments.

Since it has been suggested that early piano lessons could increase the prevalence of AP (Miyazaki, 1989), we compared the performance (Figure 4A) and the mean absolute deviation (Figure 4B) according to student's instrument modality. One-way ANOVA showed no differences in either parameters [performance: *F*_(5, 194)_ = 1.2; *p* = 0.33; mean absolute deviation: *F*_(5, 194)_ = 1.6; p = 0.16].

### Increased AP prevalence among high proficiency musicians

**Figure 5:**
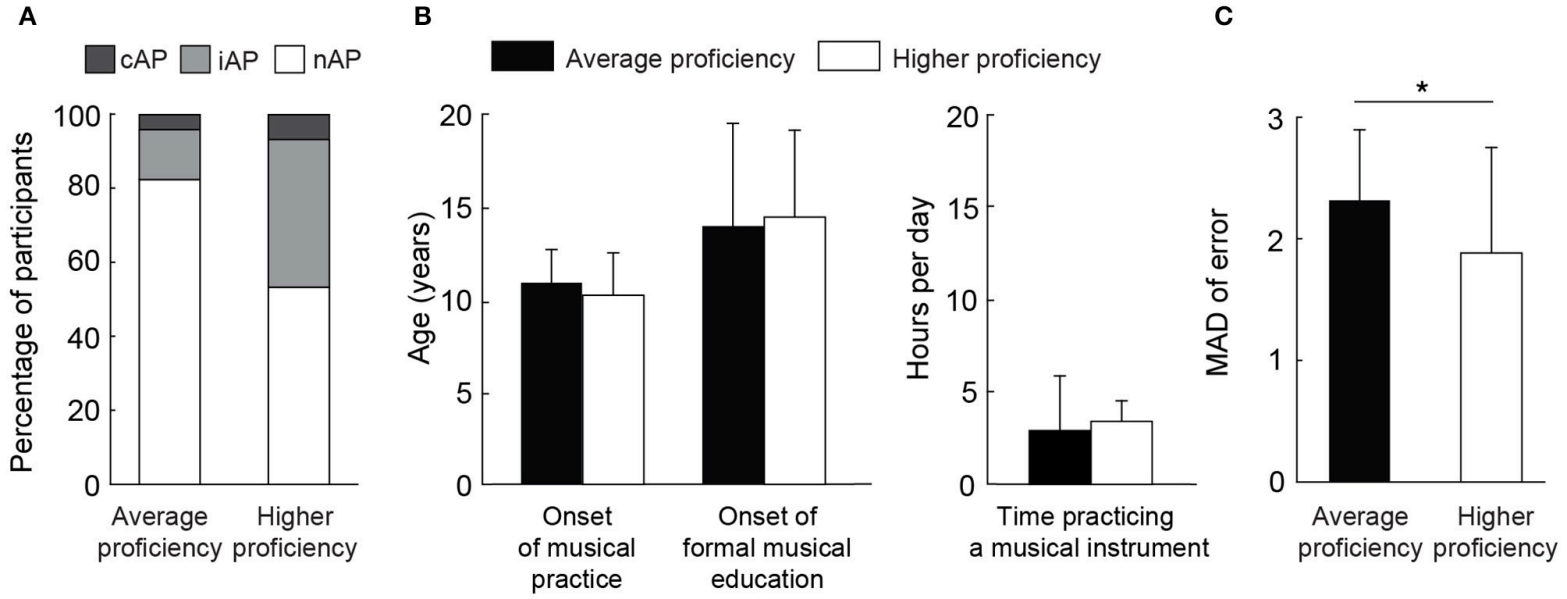
**Orchestra musicians presented a higher prevalence of AP and an improved performance in pitch-naming task. (A)** The prevalence of AP was higher in orchestra participants (higher proficiency) in comparison to regular students (average proficiency) of a music conservatory, irrespective of the threshold used to identify AP (i.e., iAP or cAP). **(B)** The onset of musical practice and the onset of formal education did not differ between both populations, as the informed averaged hours of study per day in the last year. **(C)** Mean absolute deviation of correct responses between average proficiency and high proficiency groups. ^*^p < 0.05.

To examine how music proficiency influences the prevalence of AP at the population level, we performed the same experiment in a subpopulation of the music conservatory. This second experiment included musicians who were selected to be part of the conservatory orchestra (see Section Materials and Methods). The same thresholds used to separate the regular students were applied here. We observed a higher prevalence of AP in the high proficiency group (cAP = 7%, iAP = 40%) in comparison to the average proficiency group (cAP = 4%, iAP = 14%, Figure 5A). Yet, both groups of music students did not differ in their musical history or recent daily practice, as shown by the onset of musical practice [*t*_(47)_ = 0.29, *p* = 0.77, *t*-test], the onset of formal instruction [*t*_(47)_ = 0.09, *p* = 0.92, *t*-test], and the number of hours in their daily practice in the last year [*t*_(47)_ = 0.82, *p* = 0.41, *t*-test; Figure 5B]. Excluding the correct responses (i.e., absolute deviation = 0), students from high proficiency group guessed more closely the stimuli than average proficiency group, as revealed by the smaller mean absolute deviation of the high proficiency group [*t*_(45)_ = 2.02, *p* < 0.05, *t*-test; Figure 5C]. The effect size was estimated to be 0.5 (Glass's Δ method).

## Discussion

Here we present the first quantitative investigation of AP ability in Latin America using traditional psychophysical methods. Previous work, which only employed self-report questionnaires to assess AP, described a prevalence of 5% among Brazilian conservatory students (Germano et al., 2011, 2013). This prevalence is similar to the one described here using self-reported questionnaire (3.5%; Table 1). However, some individuals who reported being AP possessors did not reach the threshold criteria in the pitch-naming test (4 out of 7). Anecdotal reports suggest that musicians covet AP ability, although the reasons for such aspiration are undefined. AP has been associated with both improved musical transcription capability (Dooley and Deutsch, 2010) and impaired interval recognition (Miyazaki, 1995). A possible crave for AP could have an important impact on the conclusions driven from studies using self-reported questionnaires exclusively (Gregersen et al., 2001). Here, participants knew they would take part in a pitch-naming test immediately after answering the questionnaire, which might have attenuated the false-positive rate and boosted the false-negative rate. In this report, we showed that estimations based on self-reported questionnaires and pitch identification test evaluation can differ significantly, which makes the use of standardizing psychophysical tests mandatory, as previously suggested (Bermudez and Zatorre, 2009).

Few papers have reported the pitch-naming performance distribution in the population (Athos et al., 2007; Bermudez and Zatorre, 2009; Miyazaki et al., 2012). In those that do, it is apparent that the performance in the population is actually a continuum rather than a discrete bi-modal attribute, although there may be a tendency for the formation of clusters at the extremities depending on the applied method. For example, using a web-based test Athos et al. (2007) found a distribution with a bimodal tendency; however, the lack of supervision during the test may be crucial, because there is no guarantee that participants did not adopt any reference before each stimuli block. This would allow the use of other skills, such as relative pitch, or even the use of an instrument that can give some feedback to the participant, which would increase participants' performance, especially those with intermediate performances. Another factor that can influence participants' performance is the way they are recruited, as early discussed by Bermudez and Zatorre (2009). In this sense, we used a sampling method that does not allow the self-selection and that, despite being applied in groups, occurred under the supervision of experimenters, ensuring that there is no external reference available to the participants—an indispensable condition for measuring AP.

One key aspect when investigating the prevalence of AP is the inclusion criteria and the threshold used to determine whether the participant has the ability. In most studies, only a percentage of correct responses in a given number of trials combined with an arbitrary threshold to identify AP possessors is used (Deutsch et al., 2006). Conservative studies define a threshold spanning from 85 to 100% of correct responses (Miyazaki, 1989, 1990; Deutsch et al., 2009, 2006), while inclusive approaches use smaller thresholds. For example, some authors would consider evidence of AP when performance is simply above chance (Oechslin et al., 2010). As expected, this approach yields a higher number of individuals with AP. Also, some studies allow one or even two semitone distance from the presented stimuli as a correct response (Keenan et al., 2001; Loui et al., 2011). Such approaches include various levels of AP, which decreases the possibility of identifying and distinguishing different types of AP. In the present work, we applied a validated pitch identification test (Deutsch et al., 2006) in the classroom of the music conservatory, and observed a prevalence of 4% (conservative threshold) or 18% (inclusive and conservative thresholds summed; Figures 1A,B). These values are in accordance with previous findings in conservatory and occidental populations (Baharloo et al., 1998; Deutsch et al., 2006; Miyazaki et al., 2012). Together, these results demonstrate that AP is not as rare as previously suggested (Bachem, 1955; Takeuchi and Hulse, 1993), when considering a population of music students.

To further investigate whether the iAP group is in fact a set of some type of AP possessors, we analyzed the mean absolute deviation of incorrect answers (Figure 1E) and it revealed that the iAP group is more accurate in their responses than the nAP group (which responded as expected by chance). This result, together with the statistical method used here for the detection of AP possessors, reinforce the idea that, despite not presenting high performance in pitch identification task, the iAP group has some ability of absolute pitch perception.

### Pitch class and the label preference effect

Most studies that addressed the performance according to pitch class were done in AP possessors only, although subject's classification and their levels of performance varies significantly between studies. Regardless, all reports reviewed here found that white-key notes were more accurately and faster identified than black-key notes (Miyazaki, 1988, 1989; Takeuchi and Hulse, 1991; Athos et al., 2007; Bermudez and Zatorre, 2009). In our study, no significant difference in white-key vs. black-key notes performance was found in cAP group; however, a clear white-key note preference was observed in both iAP and nAP groups (Figure 2). Although, the reasons for pitch class preference in iAP group can be speculated, it would be difficult to extend this explanation for the nAP group, whose performance was below chance. We believe that pitch class preference can be explained by several factors. One of them is the *perceptual magnet effect* as suggested earlier by Athos et al. (2007). The hypothesis behind this concept states that the inability to distinguish similar sounds as belonging to different categories would result in naming auditory stimuli into one single prototypic label. In fact, nAP and iAP groups were more likely to write down a white-key than a black-key note when compared to the expected white-key and black-key proportion in the chromatic scale (Figure 2C). Another factor is the leaning and readiness for the participant to write down notes without mistake, which increases the likelihood of making correct choices by chance. Additionally, the specific note with the highest performance in nAP group was the “C,” which turns to be the reference pitch for most students when they start learning the major scale. All these factors argued previously, combined with the fact that the pitch-class effect was present in nAP group (Figure 2A), reveal that non-acoustic features, as label familiarity (e.g., historical reference notes) and pitch symbolic representation (e.g., presence or absence of accidents—sharp or flat), can influence pitch performance. This suggests that the road toward true (genuine) AP ability involves refraining cultural pitch preferences.

### Factors that influence absolute pitch ability

There is evidence suggesting that playing a musical instrument, especially in the initial years of musical training, the *sensitive* age (Vitouch, 2003; Deutsch et al., 2009, 2006, 2013), may influence the development of AP (Miyazaki, 1989, 1990; Miyazaki and Ogawa, 2006). We tested this hypothesis in our work, but found no significant result when we measured the performance of the volunteers in the pitch-naming test in relation to the modality of the instrument (Figure 4). It has been suggested that the sensitive period would span from 3 to 7 years old (Takeuchi and Hulse, 1993; Baharloo et al., 1998; Deutsch et al., 2009), and in the present work, we observed that the earlier a musician started practicing music, the higher was the prevalence of AP (Figure 3). However, the age of onset referred here is twice the one described in previous publications (Miyazaki et al., 2012; Deutsch et al., 2013). One possible explanation for such discrepancy may be related to cultural differences, where Brazilian music students often start to learn and practice music by playing “by ear” with friends and/or family instead of formal enrollment at a music school. Interestingly, the prevalence of AP rose significantly when considering the onset of formal music education (Figure 3B). Finally, intriguing evidence suggests that early training may be neither necessary nor sufficient to the full expression of AP ability (Brown et al., 2003).

Considering the higher age of onset of musical training in our sample of AP possessors (in comparison to previous reports), one may ask whether the neurobiology of the AP reported here is qualitatively different from the AP developed during the sensitive period. Early and recent studies have proposed the existence of different strategies of AP perception, named pseudo-absolute pitch and quasi-absolute pitch (Bachem, 1937; for a review see Levitin and Rogers, 2005). In those cases, linking pitch memory to pitch labeling would be less automated and more cognitively demanding than in individuals who acquired the ability early in life. Future studies are needed to determine whether different strategies yielding absolute pitch perception in fact exist. Possible methods should attempt to categorize the performance of participants using varied forms of stimuli (e.g., musical and non-musical sounds with different spectral statistics), while taking into account other physiological markers, such as reaction time, heart rate, skin conductance, movement-related EMG and EEG evoked potential (Itoh et al., 2005).

Language has also been proposed to influence musical abilities (Elmer et al., 2013, 2014) and the development and expression of AP (Rakowski and Miyazaki, 2007; Deutsch, 2013). It has been suggested that linguistic experience early in life can alter phonetic perception (Kuhl et al., 1992), and such environmental constrain would facilitate the development of AP ability (Deutsch et al., 2004). It is generally assumed that Portuguese comprehension does not depend on tonal interpretation (Marques et al., 2007), despite regional accents, which can vary significantly in different parts of the world to produce rich melodic speech (Fernandes, 2007). In addition, prosody can play an important role in social communication adding complex layers of interpretation to the daily conversation; but prosody does not define the meaning of any spoken word when in isolation, as it does for tonal languages (Yip, 2002). Therefore, we believe that the melodious accent of the Brazilian language did not contribute to the prevalence of AP among Brazilian musicians. In this respect, AP prevalence was found to be higher in China, a tonal language country, in comparison to United States conservatories (Deutsch et al., 2006). This result was later confirmed by another study that observed a higher prevalence of AP only among students who speak a tonal language fluently in one music school in the United States (Deutsch et al., 2009). These observations are in agreement with a previous non-controlled, questionnaire-based survey, which found high prevalence of AP among Asian students (Gregersen, 1999). However, another large-scale experiment with similar methodology compared the prevalence in music schools in Japan and Poland, and found a higher prevalence in Japanese students-who do not speak a tonal language (Miyazaki et al., 2012). The authors argue that learning methodologies can better explain the differences in AP prevalence, which raises the question about the role of music proficiency in the expression of AP.

### The influence of music proficiency

Musicality can be hard to define (Sloboda, 2008), despite bona fide attempts to create a framework capable of measuring it (Seashore, 1915; Levitin, 2012). Musical assessment tests appraise patches of music proficiency in the real world and consequently, scores can be highly dependent on the performance in specific features, as perception (time and melody) or sensory-motor integration (sight-reading). Thus, these tests do not provide the opportunity for musicians to exhibit their proficiency as a whole, and therefore, important aspects of musicality are neglect, for example, creativity, expression, or emotional communication (Levitin, 2012). Here, to investigate the relationship between proficiency and absolute pitch ability, we compared two populations of musicians of the same school, the regular students and those selected to take part in the Music School Symphony Orchestra. As mentioned early, those students comprise a special group of musicians who are selected by a tough orchestral competition, in which various aspects related to music proficiency are evaluated, such as execution, interpretation, technique, and sight-reading.

In the orchestra group, we observed that AP prevalence was higher (cAP = 6.7%, iAP = 40%) than in the average proficiency group (cAP = 4%, iAP = 13.5%; Figure 5A). This result suggests that musical proficiency is associated with increased performance in a pitch-naming test. However, this result is neither explained by the onset of musical training or formal music education, nor by how much participants practice music daily. Moreover, it is not possible to assert causality between proficiency and AP prevalence. In the same way that intense training could lead to higher pitch identification performance, AP possessors could become high performance musicians easily. Further studies are necessary to unveil whether musical proficiency leads to AP, or AP leads to higher proficiency, or both phenomena are consequences of a common cause. Several studies showed correlations between musical proficiency and other complex abilities, such as speech perception (Schön and François, 2011; Christiner and Reiterer, 2013) and mathematical performance (Schmithorst and Holland, 2004). Interestingly, in all these studies the correlations are associated with an enhanced working memory. Not surprisingly, there is evidence that AP is associated with an increased capacity of auditory digit span, i.e., the ability to memorize and recall a specific sequence of numbers (Deutsch and Dooley, 2013). In this way, it was recently shown that individuals with high working memory capacity are better in learning/improving discrimination of pitch categories (Van Hedger et al., 2015). It is possible that an increased working memory capacity is the common link between musical and other non-musical abilities, in the same way that the prevalence of AP and higher musical proficiency seem connected. Still, further studies are needed to disentangle the participation of music proficiency and increased capacity of working memory in AP development.

## Conclusions

Using a combination of questionnaire and a pitch-naming task, we were able to quantitatively measure AP prevalence in a population of Brazilian musicians for the first time. In addition, we demonstrated a white-key preference over black-key notes in subjects without AP ability, which suggests that this preference is not related to acoustic properties of pitch. Despite our volunteers started musical practice later than reported in previous studies, it was possible to confirm that there is a relationship between the prevalence of AP and the age of onset of musical practice. This suggests that the interaction between plasticity and training is more complicated than the simple opening and closing window of the sensitive period. Thus, we speculate that probably there are many different ways to achieve some degree of absolute pitch ability. Furthermore, using a more natural method of measuring musical proficiency (admission to a fine symphony orchestra instead of the standard quantitative protocols) and a pitch identification test, we identified an association between musical proficiency and AP. However, further studies are necessary to unveil the role of different variables that may contribute to this correlation, such as working memory.

## Author contributions

RL and CQ conceived and designed the experiments and analyzed the data. RL and SM performed the experiments. RL, SM, and CQ wrote the manuscript.

### Conflict of interest statement

The authors declare that the research was conducted in the absence of any commercial or financial relationships that could be construed as a potential conflict of interest.
